# Flexibility of emerging face categorization at different levels of abstraction

**DOI:** 10.1167/jov.21.5.22

**Published:** 2021-05-19

**Authors:** Miguel Granja Espírito Santo, Johan Wagemans

**Affiliations:** 1 Department of Brain and Cognition, KU Leuven, Belgium

**Keywords:** categorization, object recognition, visual feature, visual appearance, global and local aspect

## Abstract

Categorization of visual stimuli at different levels of abstraction relies on the encoding of relevant diagnostic features present at different spatial scales. We used the Eidolon Factory, an image-manipulation algorithm that introduces random disarray fields across spatial scales, to study how such a process flexibly combines perceptual information to perform successful categorization depending on task demands. Images of animal faces, human faces, and everyday objects were disarrayed coherently (random fields correlated) or incoherently (random fields randomized) to create a family of 50 eidolons per stimulus image with increasing disarray. Participants (*N* = 243) viewed each family of eidolons in a smooth sequence from maximum disarray to no disarray and performed a category verification task either at the superordinate (any face type) or basic (human face only) levels at two levels of uncertainty: participants in one group used their gut feeling to respond, whereas another group had to be sure of their decision. When participants used their gut feeling to respond, we observed a superordinate-level advantage. When they were sure of their response, we observed a basic-level advantage. Coherently disarrayed sequences impaired target detection compared to incoherently disarrayed sequences for both levels of response certainty. Furthermore, participants’ sensitivity in the Any Face condition increased when they observed coherently disarrayed sequences and had to be sure of their response. These results suggest that the visual system does not strictly adhere to feedforward processing but flexibly adjusts to the relevant perceptual information depending on task context.

## Introduction

To understand the visual input that is constantly bombarding perceivers, categorization is a crucial process in making sense of the stimuli by parsing out what is important and what is not to be able to group stimuli in a particular category or to put them in a different category. Flexibly adjusting to the demands of the environment becomes essential in extracting the right level of perceptual information and matching it to the stored categorical representations in our brain. Categories can be broadly defined as a group of objects that share structural, functional, and semantic properties (Rosch, Mervis, Gray, Johnson & Boyes-Braem, 1976). Within the hierarchy of visual processing, categorization can take place at different levels of abstraction. For instance, the superordinate level is described by the most general concepts (animal), the basic level is described with more specificity (dog), followed by the subordinate level, which requires even further detail (German Shepherd). In pioneering work by Rosch and colleagues, they observed that basic-level categories were verified more quickly than those at the superordinate level (Mervis & Rosch, 1981). This effect was expanded by Grill-Spector and Kanwisher (2005), who demonstrated that as soon as the object was detected, it was categorized at the basic level. The speed advantage observed was named the “entry-level” to reflect the moment when perceptual information first makes contact with semantic knowledge (Jolicoeur, Gluck, & Kosslyn, 1984).

Results of studies using ultra-rapid presentations demonstrate a different effect, where the superordinate level categories are detected first (Besson, Barragan-Jason, Thorpe, Fabre-Thorpe, Puma, Ceccaldi, & Barbeau, 2017; Boucart, Lenoble, Quettelart, Szaffarczyk, Despretz, & Thorpe, 2016; Fabre-Thorpe, 2011; Joubert, Rousselet, Fize, & Fabre-Thorpe, 2007; Rousselet, Macé, & Fabre-Thorpe, 2003; Thorpe, Fize, & Marlot, 1996; Wu, Crouzet, Thorpe, & Fabre-Thorpe, 2015). In these types of tasks, a go/no-go paradigm is typically used, where participants view images flashed very briefly (<30 ms) and must verify whether the target is present (go) or not (no-go) of a certain predefined abstraction level. In studies reporting the basic-level advantage, on the other hand, the stimulus presentation is typically longer, or even indefinite (i.e. until response). The typical finding of the ultra-rapid presentation experiment is that superordinate level categorization (e.g. animal) appears to be faster than basic-level categorization (e.g. dog; Macé, Joubert, Nespoulous, & Fabre-Thorpe, 2009), which is counterintuitive because participants need to consider many more possible matching items at the superordinate level than at the basic level. Such findings are typically explained by positing that object recognition generally follows a coarse-to-fine processing scheme, where coarse features may be sufficient to trigger a superordinate representation but not a basic level one (Greene & Oliva, 2009; Thorpe et al., 1996). The reverse hierarchy theory can also explain the superordinate level advantage, as the feedforward sweep activates broader categorical representations first, and then later, after feedback takes effect, recurrent processing could provide more details that would be required for categorization at the basic level (Ahissar & Hochstein, 2004).

Whether the entry point is at the superordinate or basic level is of great importance to be able to understand how perceptual information extraction and processing is organized in terms of feedforward and feedback mechanisms. However, it seems that the entry point of the hierarchy is not fixed, and it can be shifted upward or downward in specificity. For example, extensive visual experience in the categorization of particular objects can erase both the superordinate and basic level advantage found in the general population without such extensive experience. Experience in detecting objects at the subordinate specificity level (e.g. different bird species for bird watchers) leads to similar or sometimes even superior response times and accuracies compared to the other levels (Mack, Wong, Gauthier, Tanaka, & Palmeri, 2009; Tanaka & Farah, 1993; Tanaka & Taylor, 1991). Accounting for the low-level feature content in the images used can also erase the superordinate level advantage. For example, during ultra-rapid scene categorization, it was found that behavioral differences across abstraction levels are the outcome of variations in perceptual discriminability between stimuli (Sofer, Crouzet, & Serre, 2015). This is supported by the finding that, in some studies, the stimuli are comprised of stock photographs that have pre-segmented backgrounds, which aids figure-ground segmentation and consequently facilitates the rapid detection of animals at the superordinate level (Wichmann, Drewes, Rosas, & Gegenfurtner, 2010). Sensitization to a specific spatial scale can also disrupt coarse-to-fine processing and bias the use of spatial information in favor of high or low spatial frequency content (Oliva & Schyns, 1997). More recently, using a label verification task with ultra-rapid stimulus presentation, Mack and Palmeri (2015) found a superordinate level advantage if trials were blocked by abstraction level. On the other hand, if the level at which the verification occurred was randomized, a basic-level advantage was observed. They argued that these contradicting results highlight a crucial component that is often missed in the literature – how local trial context influences how the brain prioritizes which diagnostic information is extracted and interpreted.

These studies highlight that categorization does not necessarily follow a strict hierarchy. One potential explanation that is often overlooked is how the correlational structure of different spatial scales interacts with task specificity and response certainty. If the coarser blobs can help predict where the edges at the finer scales are present, then the brain having learned the associations between them would be able to jump ahead in the processing stream and assume the remainder of the appearance or at least be guided by that embedded cross-scale statistical structure. If this is the case, disrupting those relationships could give us a window into how categorization relies on the perceptual information present at different scales.

**Figure 1.**
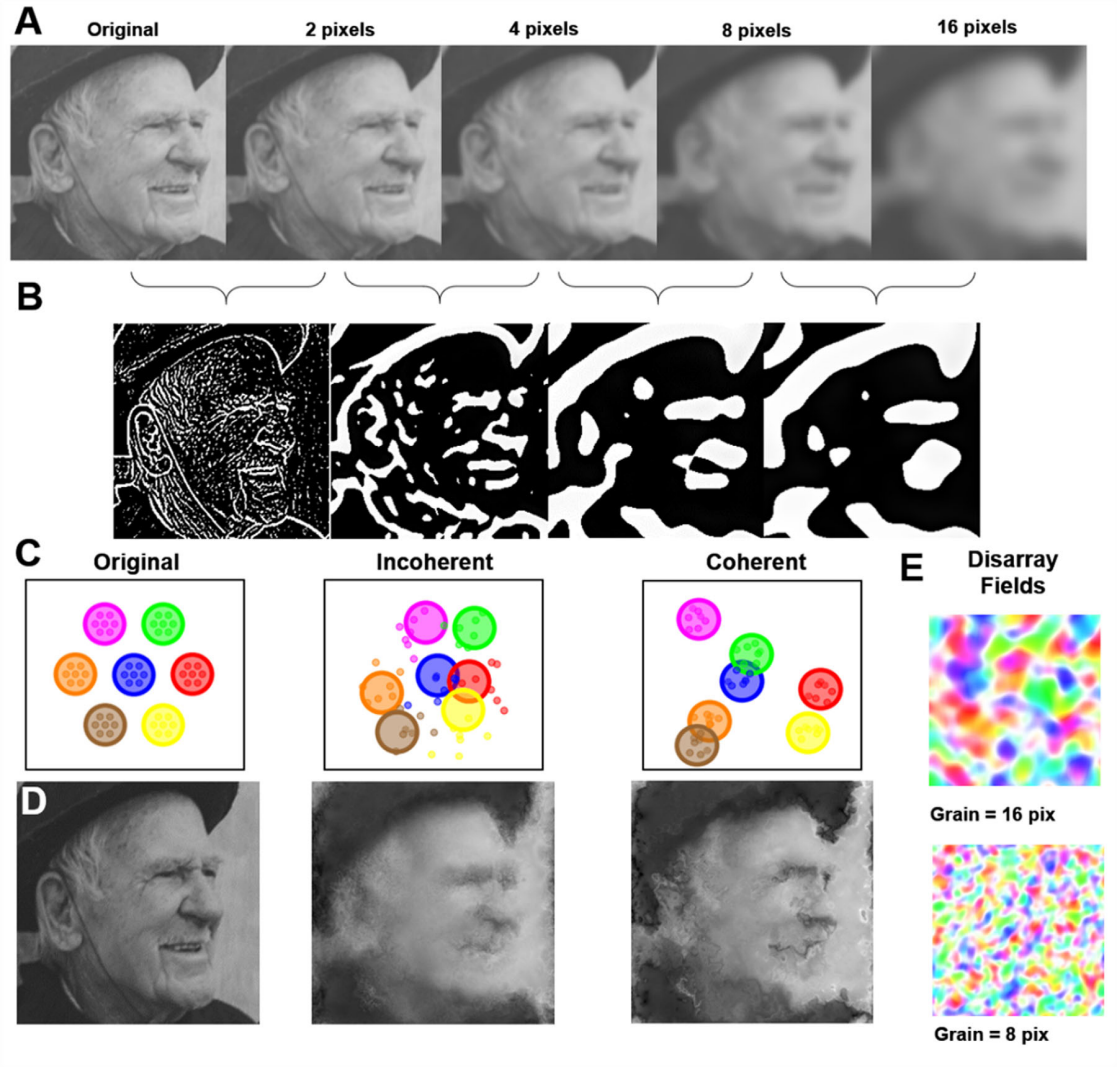
Examples of the processing steps in the Eidolon Factory using one of the stimuli presented in the experiment. In (**A**), some layers of the scale space created using a blur of 2, 4, 8, 16 pixels radius. (**B**) Shows the resulting Difference of Gaussian (DoG) layers of the original scale-space decomposition above. This yields a handle on the blobs and edges present at each spatial scale and on which we can apply the spatial pixel displacements. In (**C**), the blobs represent spatial scales of different resolutions. In the incoherent case, the reach (the spatial displacement from the original) acts independently and randomly. In the corresponding eidolon below in (**D**), the effect is seen as a diffusion of the edges in the image. In the coherent case, the finer blobs are contained within the larger blobs even if the former are individually displaced. The effect is seen in the corresponding eidolon in **D**, which results in deformations of the local structures, placing them in unexpected locations but without losing their edginess. (**E**) Shows two examples of disarrays fields with different grain sizes. The hue shows the direction of the pixel displacement. The inserts in **C** were adapted from Figure 10 in Koenderink et al. (2017). Source of original image https://face-categorization-lab.webnode.com/resources/natural-face-stimuli/.

In this study, we aimed to investigate how the encoding of relevant perceptual information changes depending on the abstraction level and response certainty of face categorization. To investigate this process, we used the Eidolon Factory (Koenderink, Falsecchi, van Doorn, Wagemans, & Gegenfurtner, 2017), an image-manipulation algorithm that introduces random disarray fields across spatial scales before recomposing the image again. *Eidolons* are the product of these manipulations. They are a family of equivalent images that change along one or more meaningful parameters that are sometimes perceived to be the same as the original image, or different, depending on whether the perceptual impressions perceivers get when looking at them share the same building blocks in terms of localized spatial-frequency content or not. The first of these parameters is the *reach*, which defines the amplitude of the pixel disarray applied to the different spatial scales. The second parameter is the *grain*, which controls the graininess or blur of the pixel disarray fields. The last parameter is the *coherence*, which dictates the extent to which the disarray field at one specific spatial scale influences (i.e. is correlated with) the disarray fields applied to the subsequent spatial scales. Figure 1 explains the parameters of the Eidolon factory visually, and more details can be found in the Stimuli section of the Methods.

In our experiment, we focused only on varying the reach and the coherence of the disarray (fully coherent or fully incoherent) of the eidolon families created, while keeping the grain of the disarray fields at the same size as the resolution of the filters used to decompose the images. This meant that at the image level, coherent and incoherent disarrays manipulated relatively more strongly either the more global or the more local structures of an image, respectively (i.e. roughly corresponding to blobs and edges, respectively). As one can see in Figure 1D, this manipulation creates images in which larger areas of the original image seem to be displaced or transformed (right) or the edges appear more blurred (middle), compared to the original image (left), respectively. For any given eidolon family sequence, coherent or incoherent disarray structures were resolved over time, allowing to explore how the different sources of perceptual information would interact with categorization speed and accuracy for different task settings. Then, to disentangle how response certainty would interact with perceptual and categorical manipulations, participants completed a category verification task either at the superordinate (any face type) or basic (human face only) levels and at different levels of uncertainty: one group of participants was instructed to use their gut feeling to respond, whereas a separate group of participants was told they had to be sure of their decision. The overall purpose of these manipulations was to achieve a more detailed understanding of the flexible use of different spatial scales and how this unfolds over time, from initial glances of meaningless image patches and pixels, to hunches of possibly meaningful structures or object parts, to definite and refined object categorization at different levels of specificity.

One of the principles of perceptual categorization, particularly for faces, is that it requires the extraction of holistic (configural) properties that arise from the relationships between the parts – and local structure like edges constitutes an important basis to create those parts (Kimchi, 2015; Rossion, 2008). When the structural integrity of those configural properties collapses because some parts deform or shift to unexpected places, the processes leading to successful categorization will be hindered. Consequently, it was predicted that coherently disarrayed sequences will make target detection (i.e. correct categorization at the prespecified target level) more difficult because there are salient features (with intact local properties) that can give misleading configural hints as to what the image appears to be. In incoherently disarrayed sequences, this perceptual faux pas is less likely to occur because only the edges appear more blurred, with no deformations or misplacements of larger features. In the incoherent disarray condition, the configural content is less impaired by the Eidolon factory at equivalent levels of reach; therefore, the extraction of meaning has to overcome fewer hurdles. Given that the literature is often contradictory regarding which abstraction level offers an advantage, and our technique of presenting images is completely different from previous studies (neither like the typical ultra-rapid presentation nor like the typical basic-level recognition studies), we refrained from making a level-specific prediction. Instead, we predicted that if there is a difference in how different sources of perceptual information are being used for a given abstraction level, then an interaction effect between task specificity and disarray coherence should be observed. Furthermore, responses were unpacked using Signal Detection Theory (SDT) to investigate the extent to which the global or local structure of an image could be differentiated from the external noise introduced by the different types of disarray fields and from the internal noise of the neural mechanisms involved (sensitivity; d’). Moreover, SDT can also reveal if the image and noise properties elicited a particular response bias depending on the abstraction level of the task (decision criterion; c). We wanted to explore both of these SDT parameters in relation to gut and sure responses in interaction with stimulus and task conditions but we had no specific predictions.

## Methods and materials

### Participants

Two hundred forty-three first-year psychology students (203 women, with mean age = 18.75 years, SD = 2.53) received course credits as compensation to take part in this study. Participants had a normal or corrected-to-normal vision, and they were excluded from the analysis if they showed accuracy levels below 33% for the group where a human face was the target, and below 66% where any type of face was the target. The experiment was approved by the Social and Societal Ethics Committee of KU Leuven (approval code: G-2019 04 1638).

### Stimuli

The stimuli for this experiment consisted of images that were disarrayed using the Eidolon Factory (Koenderink et al., 2017). This algorithm starts by decomposing an image into its scale-space difference layers (see Figure 1A) and applies local perturbations to each one of them before recomposing the image again. The perturbations are generated via random field vectors in both the X and Y dimensions that are the result of Gaussian filtering of normally distributed noise fields with receptive field size scaled with the resolution of the spatial scales or fixed at specific pixel width. The amplitude of the disarray created is regulated by the *reach*, the standard deviation of the distribution of the random disarray fields. In Figure 1C, this is represented by the distance travelled by each blob from its original position. The correlational structure of the disarray fields applied to each spatial scale is controlled by the *coherence* parameter, which is a weighted combination of the disarray fields that are common throughout the spatial scales. With a value of 0, the disarray fields across spatial scales are generated independently for each of the spatial scales at the same resolution, making edges fuzzier and less well defined. With a value of 1, it creates correlated disarray fields by applying a Gaussian filter to an initial noise field and then filtering it at the resolution of the scale-space for each of its layers. Therefore, the disarray fields at the finer spatial scales are contained within the disarrays applied to the preceding, coarser, spatial scales, which leads to the warping of the local feature structures (see Figures 1C, 1D). This makes coherence the key parameter for this study, as it controls the extent to which coarser scales dictate where the finer scales end up. The last parameter is the *grain*, the standard deviation of the filter applied to the disarray fields. With the grain, it is possible to dictate how spatially correlated disarray structure of the image is – with low grain and enough reach, the image will break into smaller parts in random directions; and with high enough grain in relation to the size of the objects present in the image, it will create an eidolon that appears deformed in any given direction.

**Figure 2.**
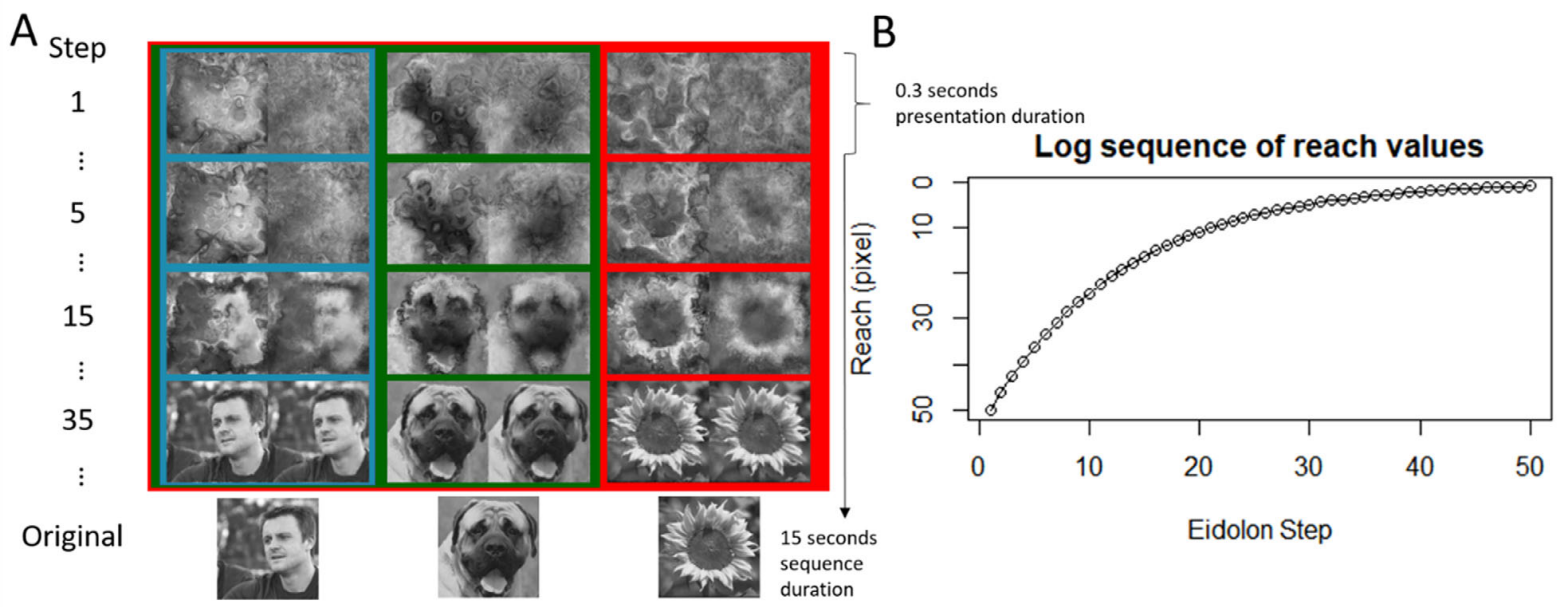
(**A**) Original stimuli, at the bottom and corresponding the eidolons generated using coherent and incoherent disarrays in different steps of reach, in two columns (left and right, respectively). The colors depict the different grouping of the categories used for the detection task. Red frame (objects) versus blue/green frames (human faces and animal faces, respectively) correspond to the superordinate group (“Any Face”), where the instruction was to verify whether any type of face was present in the eidolon family. Blue frame versus red/green frames correspond to the basic-level task (“Human Face”), where the instruction was to verify whether a human face was present in the eidolon family. (**B**) Graphical representation of the log distribution of the reach values (in pixel space). The human face image was obtained from Gao et al. (2018) and the dog and flower images were obtained from http://cbcl.mit.edu/software-datasets/serre/SerreOlivaPoggioPNAS07/index.htm.

The original stimuli used for the experiment consisted of 40 greyscale images (480 × 480 pixels) of animal faces, human faces, and everyday objects against various backgrounds and in various poses (e.g. viewpoints and positions within the image frame). They were collected from freely available image sets – namely https://face-categorization-lab.webnode.com/resources/natural-face-stimuli/ and http://cbcl.mit.edu/software-datasets/serre/SerreOlivaPoggioPNAS07/index.htm - and from Gao, Gentile, & Rossion (2018) with permission from the authors. Before applying the Eidolon Factory algorithms to these images, they were equalized for luminance and contrast using the SHINE toolbox (Willenbockel, Sadr, Fiset, Horne, Gosselin, & Tanaka, 2010). These images were then used to generate a family of eidolons with the following parameters: half of the images were disarrayed coherently (coherence = 1) and the other half incoherently (coherence = 0), creating 50 different eidolons with increasing reach from 1 to 50 pixels following a logarithmic distribution (see Figure 2). This range was selected following prior pilot experimentation. It allowed the observer to experience the stimulus sequence as gradually changing, rather than experiencing an immediate change in the appearance, as was the case with a linearly distributed decrease of the reach values. Using this logarithmic space, we could get a more refined grasp on the moments in which participants responded with different levels of certainty. The grain was proportional to the resolution of the scale-space layers generated from the image decomposition procedure. The motivation for this choice was two-fold: first, this reduced the number of parameters that would have to be tested, and second, keeping the disarray fields proportional to the resolution of the spatial scales allows for the coherent disarrays to have the desired fractal effect where the coarser spatial scales drag the finer scales with them (see the right inserts in Figures 1C, 1D).

### Procedure

Participants completed the task using an in-house online platform (https://psytests.be/). To ensure quality and timing consistency of the data, the following recommendations for optimal viewing conditions were set: monitor refresh rate of 60 Hz, monitor resolution of 1280 × 768 (or similar aspect ratio), access to a keyboard and a mouse, and the ability to do the experiment in a quiet and slightly dark environment. When these conditions were not met, participants could not start with the experiment.

Participants were directed to the online platform via a link at the end of a short questionnaire for another (unrelated) study. After logging on the online platform and consenting to participation, the experiment started with a brief description of the task and an explanation of the different certainty levels. For responses using gut feeling participants saw: “Try to respond as quickly as possible. Use your intuition or gut feeling to decide whether the image contains a face or not, even if you can't really tell yet how the whole thing looks like. The moment you get the feeling, respond immediately. So, while you are holding the space bar down, if you think, for even a moment, you can respond, STOP! - and let go of the space bar. After you let go of the space bar, you will be prompted to indicate whether the image contains the target or not. After this response, you will see the image in complete detail.” Participants that were asked to respond only when they were sure saw: “Only let go of the spacebar when you are 100% SURE that you know what the image is. It could be of any number of things: animals (cats or rabbits, etc...), or everyday objects (chairs, telephones, etc...). You don't have to hold the spacebar to the end, it's ok to let go earlier but only if you are sure. After you let go of the spacebar, you will be prompted to indicate whether the image contains the target or not. After this response, you will see the image in complete detail.” After this explanation, participants completed and eight practice trials using a set of images different from those in the actual experiment. The practice started with a reminder of which group the participant was assigned to, indicating whether the target was “Any Face” or “Human Face” and the level of certainty they should use in their responses: “Use your gut feeling,” or “Be sure of your response.” Consequently, this established a between-subjects design with four different participants groups: (2 [Gut, Sure] × 2 [Any Face, Human Face]). The two levels of response were designed to disentangle different modes of viewing of the unfolding images, reflecting a first hunch and a more definitive categorization.

At the beginning of each trial, two messages appeared instructing participants about when and how to respond, first, reminding them of what their target was and the required level of certainty (see above), and second, in a separate screen, how to respond (“HOLD DOWN the space bar when you are ready to start. Only let go when you are ready to respond.”).

While the spacebar was held down, all images (the eidolons) for a respective eidolon family were presented in descending order of reach (see Figure 2B). This way of responding was chosen because it increased the interactivity with the task and allowed for an intuitive, and perhaps more sensitive response mechanic to detecting targets. Each eidolon image was presented for 300 ms before switching to the next one in the sequence. If no other input was given, the sequence would continue to disambiguate until the 0 reach eidolon (i.e. the original image) appeared on the screen. The full, uninterrupted sequence took 15 seconds to complete. Once the spacebar was released, the stimulus sequence was interrupted, and a new question appeared on the screen asking participants “Did you see the target? Yes or No.” Responses were made using the keyboard. Once a response was given by the participants, the final, disambiguated image (i.e. the original image) was shown. In total, participants completed 60 trials each of both the coherent and incoherent disarray sequences.

### Data analysis

Data analysis was conducted using Rstudio (RStudio Team, 2018); the code and data to reproduce our results can be found at https://osf.io/pfbw5/. Participants who did not complete more than 80% of the trials (due to computer failures) or who failed to reach performance above the chance level of their given group were excluded from further analysis (*n* = 10 in total). Furthermore, we removed responses under 0.3 seconds from further analysis as this likely represents an input error or a bad response from the participant. This created a dataset of 233 participants, nicely distributed across the four groups (Any Face, Gut = 59; Any Face, Sure = 58; Human Face, Gut = 58; Human Face, Sure = 58).

A 2 × 2 × 2 ANOVA with a linear mixed-effects model (afex::mixed(); Singmann & Kellen, 2019) was conducted to investigate the effects of Disarray Coherence (coherent, incoherent) as the within-group factors and Response Certainty (Gut, Sure) and Target Specificity (Any Face or Human Face) as the between-groups factor on the reach reduced (the amount of deformation that reduced in pixels) and response times. In the analysis, only responses to human faces were included, as participants in both basic and superordinate groups would have made “yes” responses to these stimuli. The linear mixed-effects model structure was designed following the recommendations in the literature (Barr, Levy, Scheepers, & Tily, 2013; Matuschek, Kliegl, Vasishth, Baayen, & Bates, 2017). First, the maximal model structure was tested to verify convergence – in our case coherence and type were crossed with participant ID, and image ID was nested within groups. Then, if this failed, a more parsimonious model was tested until convergence was achieved. This approach was aimed at minimizing uncontrollable variability because of noise in the participant responses, due to unaccounted low-level feature variability present in the image sequences or image-specific interactions with the parameters of the Eidolon Factory. Significance evaluations were conducted using Satterthwaite approximation for degrees of freedom, which reduces the likelihood of type 1 errors when modeling the effects (Luke, 2017). Last, sensitivity (d′) and the decision criterion (c) to targets were also analyzed. Post hoc comparisons were conducted to investigate the nature of any potential interaction effect with coherence, as any other effect is outside the scope of this study. The *p* values for multiple comparisons were corrected using the Bonferroni method of adjustment.

## Results

Two separate mixed-effects ANOVA were conducted using the reach reduced before making a response and the time to lift the spacebar (response time [RT]) for stimuli containing human faces.

**Figure 3.**
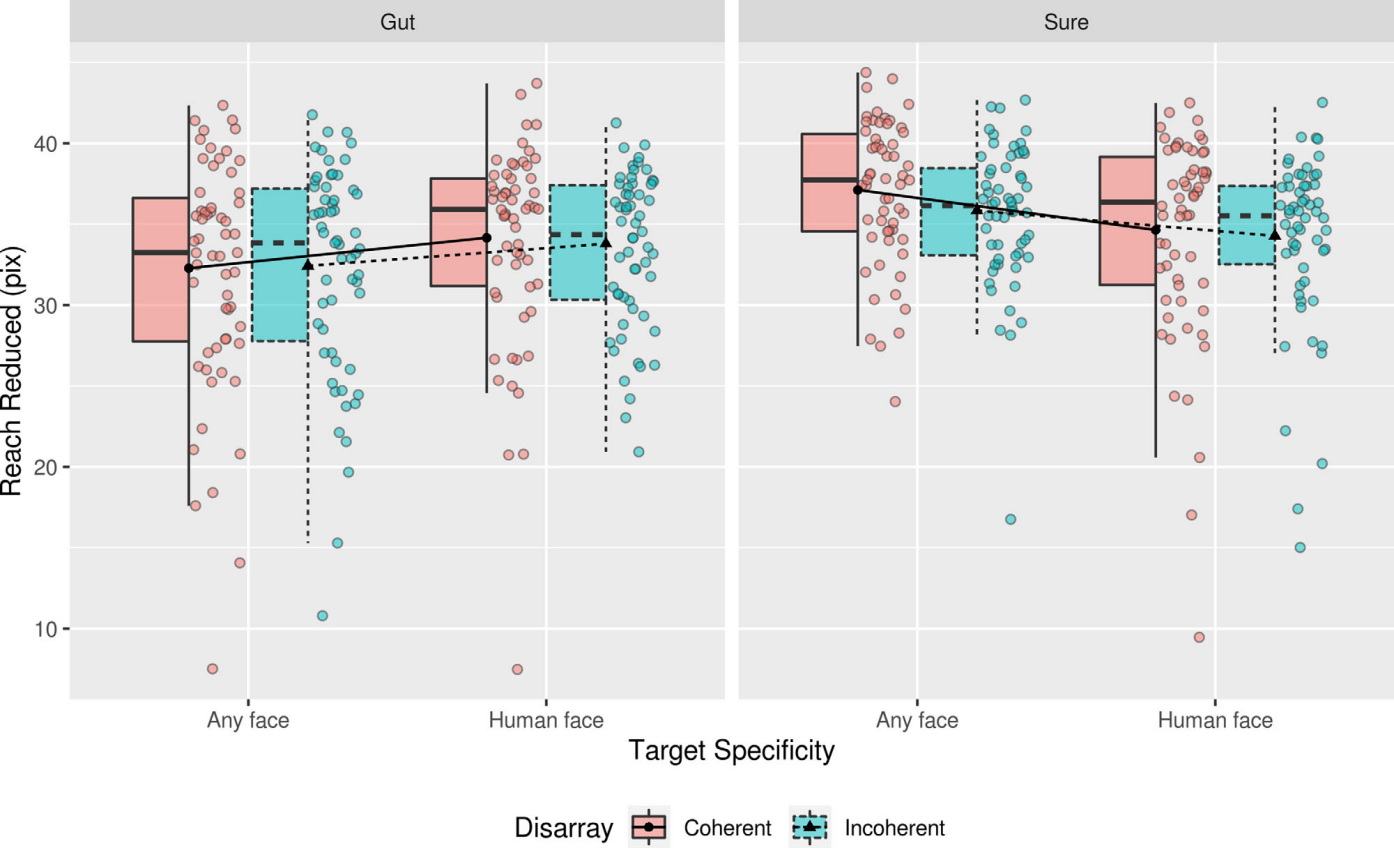
Boxplot of the estimated marginal means (EMMs) of the reach reduced in pixels for responses while using Gut feeling and being Sure of the response. This plot only depicts responses to human face stimuli in both conditions of Response Certainty. The lines connect the EMMs between the different groups for the same types of Disarray, and points represent individual participant means for each of the conditions.

The results of the mixed-effects ANOVA for the reach reduced showed a main effect of Response Certainty, *F*(1, 229.10) = 6.87, *p* < 0.01, with the estimated marginal mean (EMM) and mean difference (MD) between the different types of Response Certainty indicating that participants in the Sure condition reduced reach^1^ more than those in Gut condition (MD = 2.30, SE = 0.88). The analysis also yielded a significant interaction between Target Specificity and Response Certainty, *F*(1, 222,85) = 6.85, *p <* 0.01. From the inspection of the EMMs and Figure 3, this interaction stems from participants needing more reach to make Sure responses in the Any Face condition in comparison to those participants responding to the targets using their Gut feeling (Sure, Human Face - Gut, Any Face = 4.13, SE = 1.12). Refer to Table 1 in the Appendix to view all the other effects and EMMs.

**Figure 4.**
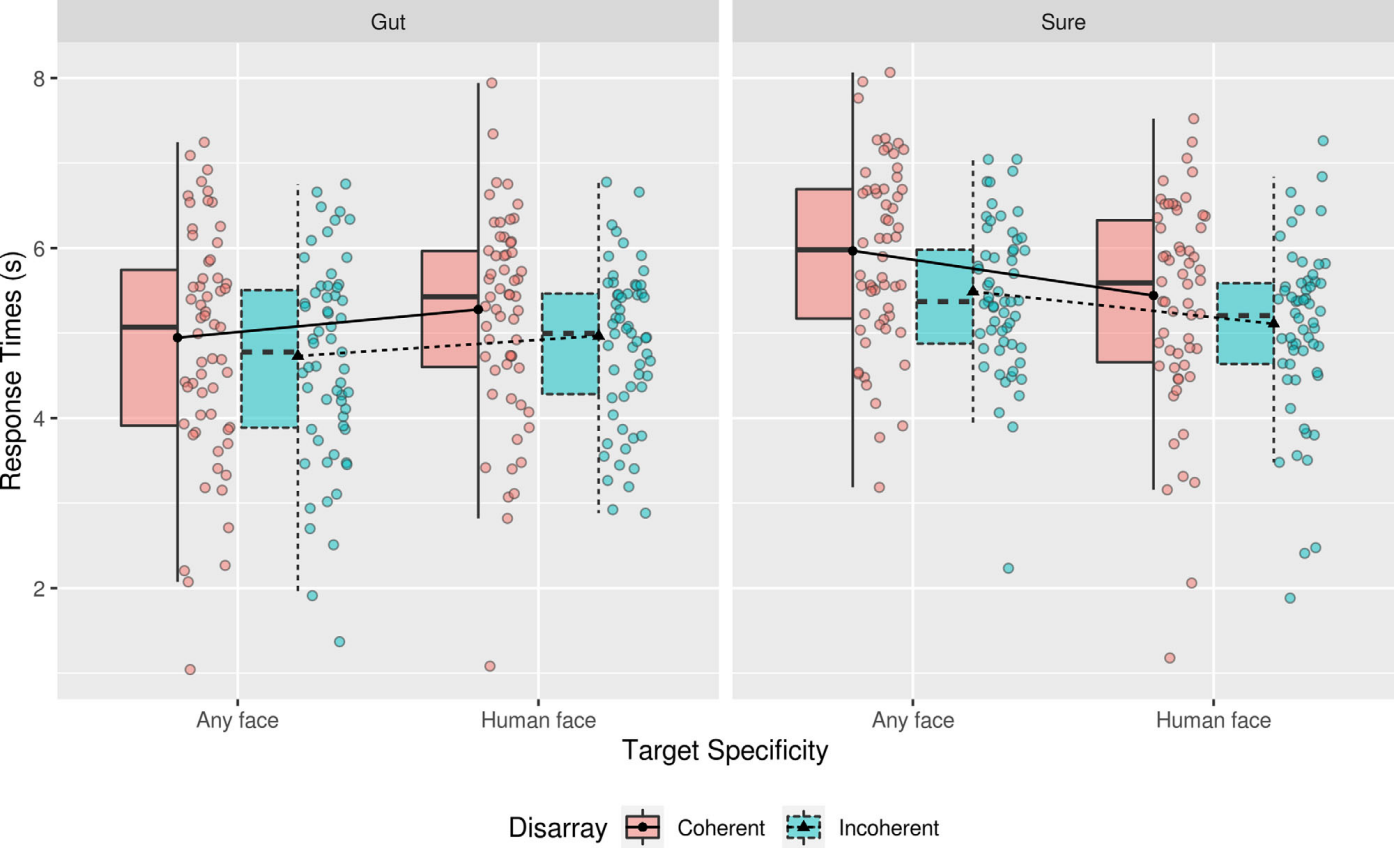
Boxplot of the estimated marginal means (EMMs) of the time to detect a target while using Gut feeling and being Sure of the response. This plot only depicts responses to Human Face stimuli in both conditions of Response Certainty. The lines connect the EMMs between the different groups for the same types of Disarray, and points represent individual participant means for each of the conditions.

The results of the mixed-effects ANOVA for response times also showed a main effect of Response Certainty, *F*(1, 173.51) = 6.51, *p* < 0.01, indicating that participants in the Sure condition took longer to detect the presence of a target than those in the Gut condition (MD = 0.89 seconds, SE = 0.24 seconds). A main effect of Disarray Coherence was also found for the RTs, *F*(1, 75.34) = 4.46, *p* = 0.04; when presented with coherently disarrayed eidolon sequences, participants took longer to detect a target (0.33 seconds, SE = 0.17 seconds). A significant interaction between Target Specificity and Response Certainty was found, *F*(1, 225.00) = 7.35, *p* < 0.01. From the inspection of the EMMs and Figure 4, this interaction follows the same direction as the one found for the reach reduced (Any Face: Sure - Gut = 0.89 seconds, SE = 0.24 seconds). Refer to Table 2 in the Appendix to view all the other effects and EMMs.

**Figure 5.**
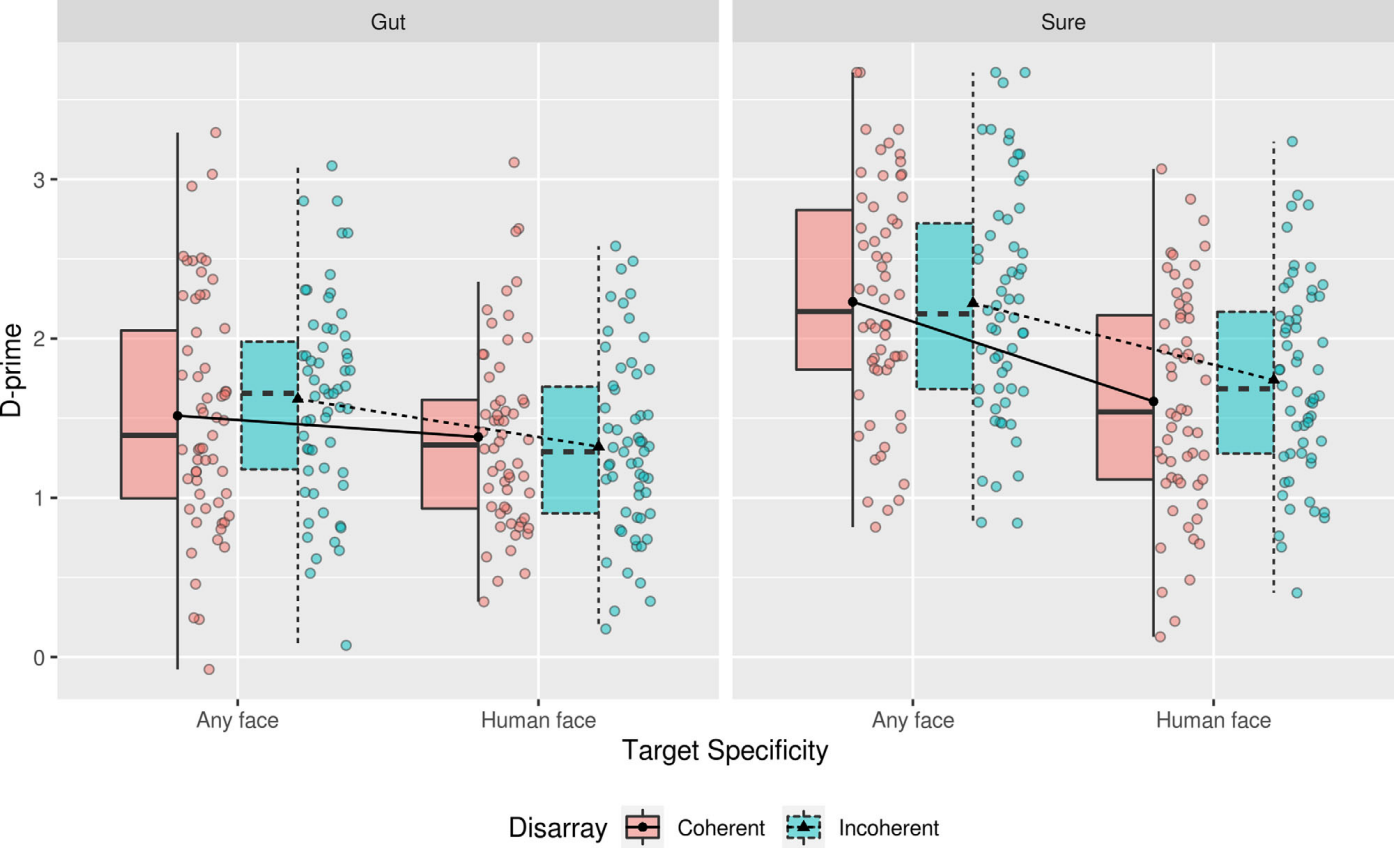
Boxplot of the estimated marginal means (EMMs) for sensitivity while using Gut feeling and being Sure of the response. The lines connect the EMMs between the different groups for the same types of Disarray, and points represent individual participant.

Results of the mixed-effects ANOVA on d-prime showed a significant main effect of Target Specificity, *F*(1, 229) = 24.43, *p* < 0.001, *η^2^* = 0.10, indicating that participants in the Any Face condition were more sensitive to the presence of a target than those in the Human Face condition (MD = 0.39, SE = 0.08); a significant main effect of Response Certainty, *F*(1, 229) = 39.47, *p <* 0.001, *η^2^* = 0.15, indicating that participants in the Sure condition were more sensitive to the presence of a target than those in the Gut response condition (MD = 0.49, SE = 0.08); a significant interaction between Target Specificity and Response Certainty, *F*(1, 229) = 4.72, *p* = 0.03. From the inspection of the EMMs and Figure 5, this interaction is caused by an increase in sensitivity to targets in the Any Face condition, while making responses at the Sure level (Any Face: Sure – Gut = 0.66, SE = 0.11; Sure: Any Face – Human Face = 0.55, SE = 0.11); and a borderline significant three-way interaction between Disarray Coherence, Target Specificity, and Response Certainty, *F*(1, 229) = 3.95, *p* = 0.048, *η^2^* = 0.02. See Figure 5.

The ANOVA conducted on the decision criterion showed no significant main effects or interactions.

Smaller 2 × 2 ANOVAs were conducted to pinpoint the source of this 3-way interaction. This analysis indicated that the source of this effect stemmed from a significant interaction between Target Specificity and Response Certainty at the level of Coherent disarrayed sequences, *F*(1,229) = 7.46, *p* < 0.01, η^2^ = 0.03. From a closer inspection of Figure 5 and the EMMs, this interaction indicated that when participants had to be sure of their responses in the Any Face condition their sensitivity to targets increased compared to participants in the other conditions (Any Face: Sure – Gut = 0.72, SE = 0.13; Sure: Any Face – Human Face = 0.63, SE = 0.13). In contrast, the incoherently disarrayed sequences showed a non-significant interaction between Target Specificity and Response Certainty was found, *F*(1,229) = 1.22, *p* = 0.27, η^2^ < 0.01. Refer to the Appendix to view all results for the other effects and the EMMS.

## Discussion

This study aimed to investigate the encoding of relevant diagnostic image features present at different spatial scales, and how it changes depending on the abstraction level of categorization and response certainty. The results showed that when participants used their gut feeling to respond, there was a superordinate advantage. When they waited until they were sure of their responses, a basic-level advantage was observed instead. Compared to incoherent disarrays, the coherent disarrays led to longer response times to detect a target, but no interaction with both response certainty or target specificity was found. The analysis of the participants’ d-prime results showed that their sensitivity to targets increased when they were responding in the Any Face condition and when they had to be Sure of their response, compared to the other conditions. An interaction between these two conditions was also found indicating that the joint effect of responding in the Any Face condition at the Sure level led to a further increase in sensitivity to targets. Furthermore, in this superordinate level condition, sensitivity to coherently disarrayed targets when employing a high level of certainty to their response was improved compared to when responding using gut feeling.

Response certainty had a consistent main effect across the measures analyzed in this task. This result indicates that participants employed different viewing modes. When using gut feeling, participants likely used a more automatic and perceptually driven strategy to respond, while they were naturally more deliberate and careful when they wanted to be sure of their response. At the main effect level, this is similar to a speed-accuracy trade-off. Waiting longer to respond inevitably leads to better performance in any type of task. However, in both reach reduced and response times, the analyses showed a cross-over interaction effect between response certainty (Gut versus Sure) and target specificity (Any Face versus Human Face). This interaction strongly suggests that a speed-accuracy trade-off is not the only process at play here. Furthermore, increased sensitivity to targets in the Any Face condition compared to the Human Face condition was also observed in both the Gut and Sure responses. For the Sure responses, this result follows a similar pattern to the one observed in the analysis of reach and response times. For Gut responses, however, participants were faster to respond while showing higher sensitivity to targets in the Any face condition. This result cannot be explained by participants employing a speed-accuracy trade-off strategy as we would expect lower sensitivity with shorter response times. Instead, it probably reflects a different response mechanism. Finally, note that it is difficult to assess speed-accuracy trade-offs or any other deliberate response strategy by directly comparing the different measures as the reach and the reaction time analyses only include correct responses, whereas the sensitivity analysis includes all types of responses.

All in all, we believe that this pattern of results highlights that the entry-level of the categorization hierarchy is not necessarily fixed. It could very well be that the default entry point occurs at the basic level, the level where perceptual information interacts with semantic knowledge or, in other words, the level where shape, semantic, and functional similarities exist (Mervis & Rosch, 1981). Our findings suggest that modulations are possible as a function of stimulus and task characteristics.

A potential explanation for this effect is that the response certainty biased what, or how much, feature information is important to discern differences between distractors and target stimuli at the different abstraction levels. For instance, we observed that while response times in both Sure and Gut levels of response are quite similar in the Human Face, in the Any Face condition the response time was considerably shorter for Gut responses compared to those at the Sure level. This was the case even though the targets in this condition are more diverse, suggesting that they share enough similarities to trigger a representation more quickly. These findings are consistent with predictions of the Parallel Distributed Processing (PDP) theory (Rogers & Patterson, 2007), which explains that the advantage of a specific taxonomic level arises not due to its privileged status, but due to factors that affect the perceptual discriminability across tasks. The PDP theory also states that, to decide the group membership of an object, its internal representation must be parsed through several semantic representational nodes that are hierarchically organized. These nodes are groups of hierarchically related “hubs” for categorical representations that have distributed activity patterns much like neuronal units. In the representational space, general semantic nodes apply across a broader group of superordinate category names, such as animate or nonanimate, and encompass items with quite different representations (in our case, face or not a face). The basic-level nodes map closely onto clusters (e.g. dog) in the representation space and consist of units of subordinate terms (e.g. Corgi, Beagle, and Labrador). The certainty of the response modulates a priori the amount of evidence needed to trigger a representation threshold. For Gut responses, the earliest extracted visual feature information in the Any Face condition shared enough similarities at the lower representational hubs to trigger a common semantic label. In turn, the higher scrutiny used for Sure level responses moves this threshold to higher representational nodes (which potentially requires different or more detailed visual information) leading to more time to detect targets. This line of explanation also fits with the hypothesis of flexible use of spatial frequency information, which suggests that different spatial scales are linked to specific stimulus types or in this case stimulus types or representational hubs (Mack & Palmeri, 2015; Mermillod, Guyader, & Chauvin, 2005; Oliva & Schyns, 1997; Vanmarcke, Calders, & Wagemans, 2016). Here, we expand previous findings by providing evidence that this flexibility also applies to different response certainties.

Compared to incoherent disarrays, coherent disarrays slowed down target detection. This effect arises probably because the coherent disarrays lose more of the global structure at higher reach while maintaining the relationship between coarse blobs and finer edges. Since this well-defined but disturbed configural structure (based on the spatial relations between the deformed and displaced parts) is more difficult to disambiguate, participants wait longer to respond. Studies that investigated how the manipulation of facial features and configurations impact facial recognition suggest that the inability to extract the configural arrangement between facial features makes a qualitative difference in how faces are processed (Goffaux, 2008; Goffaux & Rossion, 2006). The coherently disarrayed stimuli could be having a similar effect as those observed in face inversion studies. For example, both face inversion and coherent disarray image manipulations qualitatively change the representation of the incoming stimulus by rendering feature extraction more difficult due to displaced local information (Rossion, 2008).

When perceiving incoherent disarrays, the local structure is not as distracting because it slowly becomes less fuzzy and clearer at similar levels of reach compared to coherent disarrays. Furthermore, the configural content of the image can transpire through the noise at higher deformation levels, making the extraction of embedded features that give “rise” to the “whole” easier. A complementary explanation in terms of an underlying prediction mechanism also fits this suggestion. For instance, this may be related to the phenomenon of sharpness over-constancy and its proposed underlying causes (Galvin, O'Shea, Squire, & Hailstone, 1999), referring to the visual system using the information of visible edges as evidence for the presence of an edge in missing or subsequent spatial frequencies. A recent study investigating this phenomenon, also using the Eidolon Factory, reported coherence over-constancy and reach underestimation in peripheral vision, where stimuli were reported to be less distorted (less reach) and having higher coherence, demonstrating the general role of prediction in the phenomenological experience (Valsecchi, Koenderink, van Doorn, & Gegenfurtner, 2018). In this study, incoherent disarrays also allow for this prediction mechanism to occur.

The analysis of the d-prime revealed a three-way interaction between the factors of the current experiment. Figure 5 shows that the making responses at the Sure level in the Any Face condition led to an increase in sensitivity to Human Face targets. This could indicate that, because coherent disarrays slowed down the recognition process, not enough reliable edge information was available to enable a confident extraction of the meaning of the image. Because in the Human Face condition there was no such effect, this could be interpreted as a top-down modification of the cognitive parameters (degree of similarity between representations required for a match) that are compensating for having more perceptually different targets - both human and animal faces as opposed to Human Face targets only. This increases sensitivity but at the cost of time.

Future research should focus on how local image structure is involved in triggering a categorical representation. Here, we propose two potential future directions. First, it would be interesting to investigate how these eidolons could be used to trace the build-up of information from a first glance to the moment in which the categorization is triggered under conditions of rapid presentation. Second, it would be interesting to explore the electroencephalographic responses to the different types of eidolon manipulations images in a dynamic fast periodic visual stimulation (FPVS) to disentangle their respective contributions to categorization. In this paradigm, the viewers observe images of objects belonging to a wide range of categories sequentially, but at a specific presentation frequency (e.g. every sixth image) an image of a face (or any other category) would be interleaved with these distractor stimuli (Jacques, Retter, & Rossion, 2016; Quek, Liu-Shuang, Goffaux, & Rossion, 2018; Stothart, Quadflieg, & Milton, 2017). By combining our current paradigm with FPVS, in the sense that the stimulus sequences would slowly disambiguate the coherent or incoherent disarrays, it would be possible to quantify how perceptual categorization at a neural level relies on local and/or global object structure.

In sum, in this study, we set out to understand how the correlational structure of different spatial scales interacts with task specificity and response certainty. We conclude that the level of scrutiny applied to the detection of human faces interacts with the entry point in the visual hierarchy. This is exemplified by the finding that when participants were instructed to wait until they are sure of their responses, a basic-level advantage was found, but when they used gut feeling a superordinate advantage was found instead. The results also showed that coherent disarrays slowed down target detection in comparison to incoherent disarrays. The relation of these findings to coarse-to-fine processing requires further investigation. One of the problems with coarse-to-fine processing theories is the assumption that the salient features that are important for any given categorization accumulate progressively. We propose instead that top-down influences can modulate this feedforward mechanism. In addition, although we do not argue against the idea that the physical integration of visual signals *is* processed from coarse-to-fine, the visual system probably only cares about finding the right pieces of the puzzle that it is trying to complete, and it will use any number of perceptual inferences, guesses, and shortcuts to minimize any extraneous effort (Koenderink, 2019).

## Supplementary Material

Supplement 1

## Footnotes

1 The units of reach reported from here forward are all in pixels.
