# Supplementary material for: Flexibility of emerging face categorization at different levels of abstraction
Source: J Vis. 2021 May 19;21(5):22. doi: 10.1167/jov.21.5.22 (PMC8142708; doi:10.1167/jov.21.5.22)
Supplement: Supplement 1 [file jovi-21-5-22_s001.pdf]

Appendix

Miguel G.E. Santo & Johan Wagemans

Flexibility of emerging face categorization at different levels of abstraction.

Introduction

This document includes detailed tables of all the effects described in the main text.

In this experiment participants were instructed to lift the space bar when they could respond ‘yes’ or ‘no’ to whether there is a face in the sequence of ‘eidolonized’ images. There were two response certainties and two abstraction levels, which were manipulated as a between-subjects design (4 groups of participants). First, the level of certainty: this was where participants had to use their gut feeling or be completely sure about the presence of the target face in the image. Second, the level of abstraction (or Target Specificity was referred to in the main text) - participants had to let go of the spacebar when they thought they saw any type of face (human or animal) or only human faces.

Full results for the Mixed effects model using Human faces only in the Any Face condition

Table 1 - Results of the reach reduced ANOVA

	num DF	den Df	F	*p*
Coherence	1	78.544	0.615	0.435
Specificity	1	222.848	0.081	0.776
Certainty	1	229.103	6.872	0.009
Coherence:Specificity	1	205.302	0.149	0.700
Coherence:Certainty	1	78.544	0.353	0.554
Specificity:Certainty	1	222.848	6.846	0.009
Coherence:Specificity:Certainty	1	205.302	1.935	0.166

Table 2 - Results of the responses times ANOVA

	num DF	den Df	F	*p*
Coherence	1	75.338	4.456	0.038
Specificity	1	206.230	0.377	0.540
Certainty	1	173.510	6.585	0.011
Coherence:Specificity	1	35.171	0.065	0.801
Coherence:Certainty	1	75.335	0.204	0.653
Specificity:Certainty	1	224.949	7.347	0.007
Coherence:Specificity:Certainty	1	6627.801	1.602	0.206

Means for response times’ significant effects:

Certainty	Means	SE	df	lower CI	upper CI
Gut	4.980	0.142	95.610	4.698	5.263
Sure	5.502	0.145	92.306	5.213	5.790

Certainty	Means	SE	df	lower CI	upper CI
Coherent	5.408	0.129	126.615	5.153	5.663
Incoherent	5.074	0.129	126.341	4.819	5.329

Certainty	Specificity	Means	SE	df	lower CI	upper CI
Gut	Any face	4.838	0.171	160.048	4.500	5.177
Sure	Any face	5.728	0.174	154.080	5.384	6.072
Gut	Human face	5.122	0.173	162.688	4.782	5.463
Sure	Human face	5.275	0.174	155.617	4.931	5.620

Signal detection theory results

Table 3 - Results of the d-prime ANOVA

		df	MSE	F	η_p^2	*p*
Specificity	1	229	0.708	24.433	0.096	0.000
Certainty	1	229	0.708	39.471	0.147	0.000
Specificity:Certainty	1	229	0.708	4.717	0.020	0.031
Coherence	1	229	0.174	1.195	0.005	0.275
Specificity:Coherence	1	229	0.174	0.022	0.000	0.883
Certainty:Coherence	1	229	0.174	0.269	0.001	0.605
Specificity:Certainty:Coherence	1	229	0.174	3.955	0.017	0.048

D-prime means for significant effects:

Specificty	Means	SE	df	lower CI	upper CI
Any face	1.897	0.055	229	1.788	2.006
Human face	1.512	0.055	229	1.403	1.620

Certainty	Means	SE	df	lower CI	upper CI
Gut	1.459	0.055	229	1.351	1.568
Sure	1.949	0.055	229	1.841	2.058

Certainty	Specificity	Means	SE	df	lower CI	upper CI
Gut	Any face	1.567	0.078	229	1.414	1.721
Sure	Any face	2.226	0.078	229	2.073	2.380
Gut	Human face	1.351	0.078	229	1.198	1.505
Sure	Human face	1.672	0.078	229	1.518	1.826

Coherence	Specificity	Certainty	Means	SE	df	lower CI	upper CI
Coherent	Any face	Gut	1.515	0.087	334.724	1.345	1.686
Incoherent	Any face	Gut	1.620	0.087	334.724	1.449	1.790

Coherence	Specificity	Certainty	Means	SE	df	lower CI	upper CI
Coherent	Human face	Gut	1.382	0.087	335.529	1.210	1.553
Incoherent	Human face	Gut	1.321	0.087	335.529	1.150	1.493
Coherent	Any face	Sure	2.231	0.087	335.529	2.060	2.402
Incoherent	Any face	Sure	2.222	0.087	335.529	2.051	2.393
Coherent	Human face	Sure	1.605	0.087	335.529	1.434	1.776
Incoherent	Human face	Sure	1.739	0.087	335.529	1.567	1.910

Table 4 - Results of the criterion ANOVA

		df	MSE	F	η_p^2	p
Specificity	1	229	0.164	3.135	0.014	0.078
Certainty	1	229	0.164	0.075	0.000	0.784
Specificity:Certainty	1	229	0.164	1.592	0.007	0.208
Coherence	1	229	0.050	2.968	0.013	0.086
Specificity:Coherence	1	229	0.050	0.004	0.000	0.949
Certainty:Coherence	1	229	0.050	1.600	0.007	0.207
Specificity:Certainty:Coherence	1	229	0.050	0.246	0.001	0.621

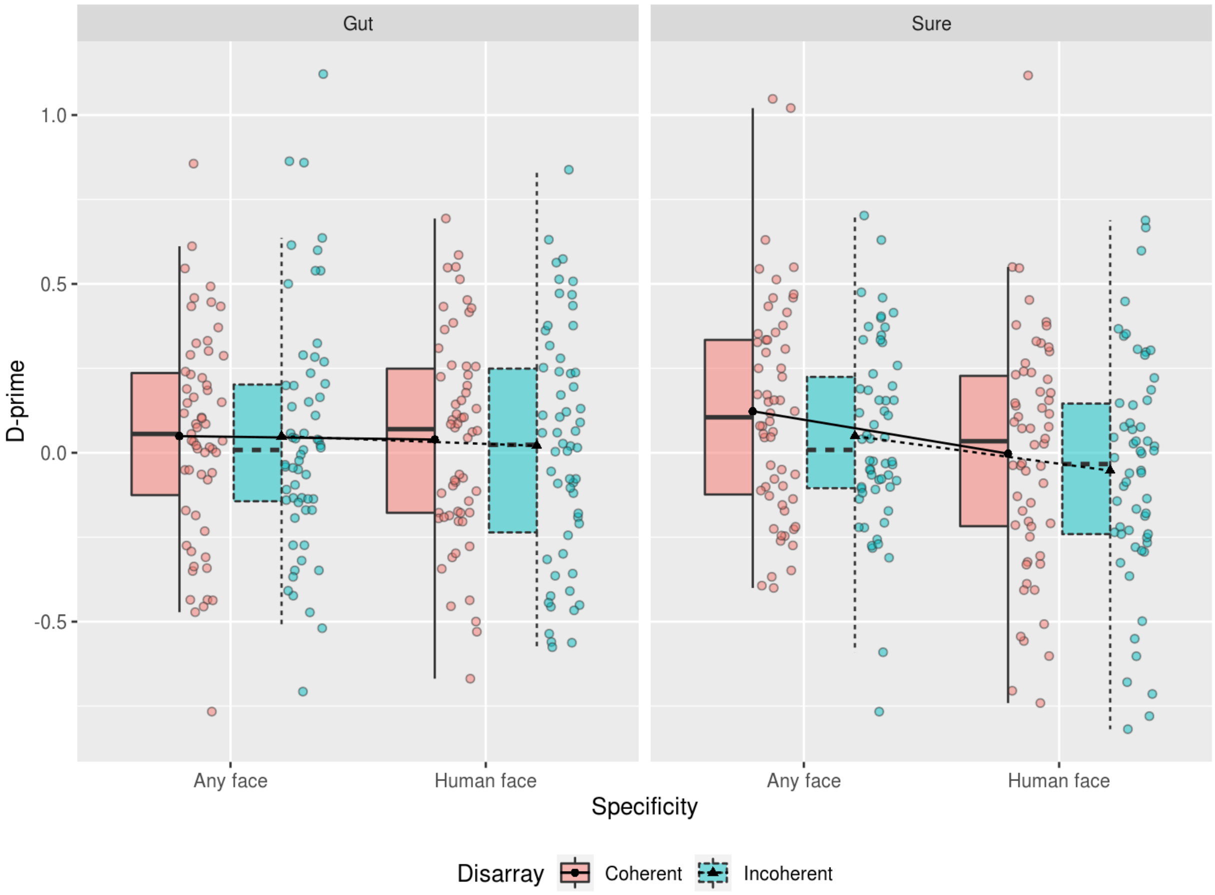

Breaking down the three-way interaction in dprime ANOVA

Table 5 - Only human face targets in the Any Face group - Anova - 2x2 (Coherence x Certainty)

	df	MSE	F	η_p^2	p

		df	MSE	F	η_p^2	*p*
Certainty	1	115	0.787	32.288	0.219	0.000
Coherence	1	115	0.209	0.646	0.006	0.423
Certainty:Coherence	1	115	0.209	0.906	0.008	0.343

Interaction non-significant

Table 6- Only human face targets in the Human Face group - Anova - 2x2 (Coherence x Certainty)

		df	MSE	F	η_p^2	*p*
Certainty	1	114	0.628	9.481	0.077	0.003
Coherence	1	114	0.140	0.556	0.005	0.458
Certainty:Coherence	1	114	0.140	3.907	0.033	0.050

Non-singifcant.

Table 7 - Only human face targets in the Gut feeling group - Anova - 2x2 (Coherence x Specificity)

		df	MSE	F	η_p^2	*p*
Specificity	1	115	0.660	4.133	0.035	0.044
Coherence	1	115	0.143	0.202	0.002	0.654
Specificity:Coherence	1	115	0.143	2.799	0.024	0.097

Interaction non-significant

Table 8- Anova Only human face targets in the Sure response group - 2x2 (Coherence x Certainty)

		df	MSE	F	η_p^2	*p*
Specificity	1	114	0.756	23.609	0.172	0.000
Coherence	1	114	0.206	1.094	0.010	0.298
Specificity:Coherence	1	114	0.206	1.427	0.012	0.235

Interaction non-significant

Table 9- Anova Only human face targets in the Coherent stimuli - 2x2 (Certainty x Specificity)

		df	MSE	F	η_p^2	*p*
Specificity	1	229	0.473	17.732	0.072	0.000
Certainty	1	229	0.473	27.147	0.106	0.000
Specificity:Certainty	1	229	0.473	7.460	0.032	0.007

Interaction Significant. Also see means for comparison.

Table 10- Anova Only human face targets in the Incoherent stimuli - 2x2 (Certainty x Specificity)

		df	MSE	F	η_p^2	*p*
Specificity	1	229	0.473	17.732	0.072	0.000
Certainty	1	229	0.473	27.147	0.106	0.000
Specificity:Certainty	1	229	0.473	7.460	0.032	0.007

Interaction non-significant

Using both Animal Faces and Human Faces in the Any Face condition.

In the analysis above, only responses to human faces were included. To investigate whether there striking difference between using responses to both animal faces and human faces, we conducted the same type of analyses.

Table 11 - Results of the reach reduced ANOVA

	num DF	den Df	F	*p*
Coherence	1	237.895	0.461	0.498
Specificity	1	232.541	1.376	0.242
Type	1	322.435	2.210	0.138
Certainty	1	286.245	16.278	0.000
Coherence:Specificity	1	86.439	0.669	0.416
Coherence:Type	1	309.410	0.004	0.950
Specificity:Type	1	84.022	4.800	0.031
Coherence:Certainty	1	234.784	0.136	0.712
Specificity:Certainty	1	231.161	5.861	0.016
Type:Certainty	1	929.646	0.090	0.764
Coherence:Specificity:Type	1	83.485	0.630	0.429
Coherence:Specificity:Certainty	1	14084.896	0.018	0.893
Coherence:Type:Certainty	1	944.024	0.008	0.930
Specificity:Type:Certainty	1	15521.401	3.780	0.052
Coherence:Specificity:Type:Certainty	1	15280.108	2.383	0.123

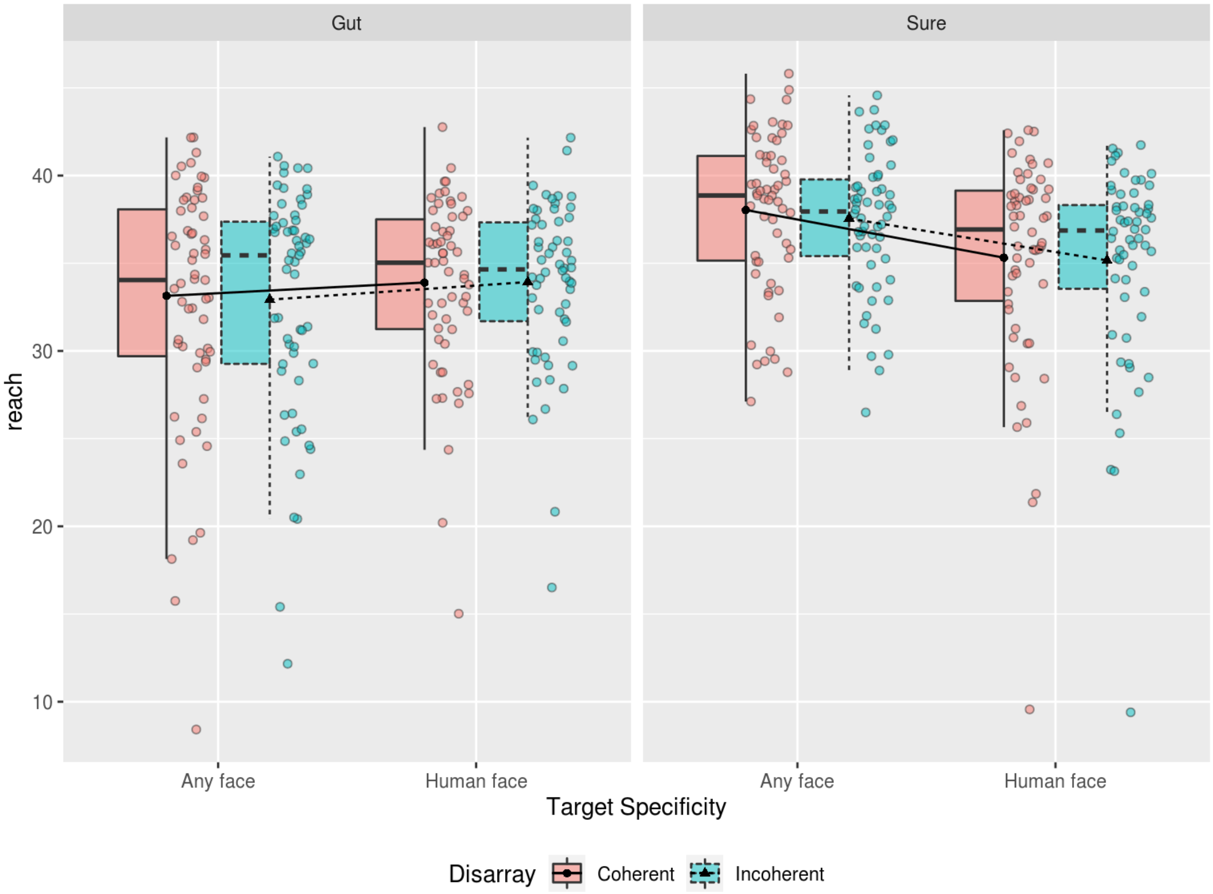

Here we see that results for both reach stopping point and response time follow the same direction as the analysis when using only human faces. The analysis shows the same significant interaction effect on both reach stopping point and RT.

Table 12 - Results of the response times ANOVA

	num DF	den Df	F	*p*
Coherence	1	109.079	4.548	0.035
Specificity	1	250.164	3.213	0.074
Certainty	1	235.495	25.331	0.000
Type	1	181.508	1.429	0.233
Coherence:Specificity	1	89.891	1.503	0.223
Coherence:Certainty	1	98.958	1.813	0.181
Specificity:Certainty	1	231.610	7.107	0.008
Coherence:Type	1	175.831	0.401	0.527
Specificity:Type	1	84.387	5.860	0.018
Certainty:Type	1	139.759	7.116	0.009
Coherence:Specificity:Certainty	1	107.283	0.525	0.470
Coherence:Specificity:Type	1	84.015	1.036	0.312
Coherence:Certainty:Type	1	139.381	0.073	0.788
Specificity:Certainty:Type	1	129.883	5.884	0.017
Coherence:Specificity:Certainty:Type	1	129.301	0.878	0.350

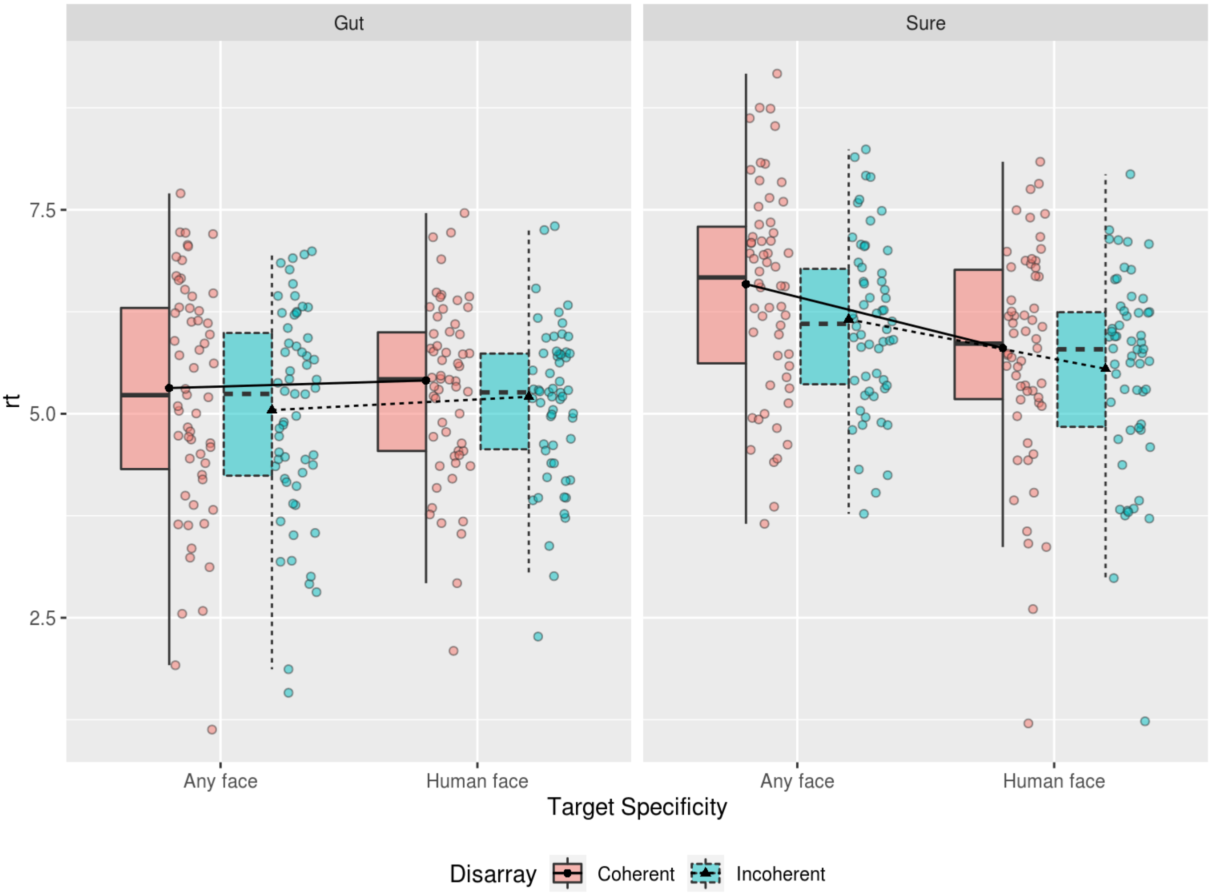

Here we see that results for both reach stopping point and response time follow the same direction as the analysis when using only human faces. The analysis shows the same significant interaction effect on both reach stopping point and RT.

Signal detection theory results using both types of target

Breaking down the three-way interaction in dprime ANOVA

Table 13 - Only human face targets in the Any Face group - Anova - 2x2 (Coherence x Certainty)

		df	MSE	F	η^2_p	p
Certainty	1	115	0.766	26.111	0.185	0.000
Coherence	1	115	0.157	1.114	0.010	0.294
Certainty:Coherence	1	115	0.157	0.738	0.006	0.392

Interaction non-significant

Table 14- Only human face targets in the Human Face group - Anova - 2x2 (Coherence x Certainty)

		df	MSE	F	η^2_p	p
Certainty	1	114	0.628	9.481	0.077	0.003
Coherence	1	114	0.140	0.556	0.005	0.458
Certainty:Coherence	1	114	0.140	3.907	0.033	0.050

Interaction non-significant

Table 15 - Only human face targets in the Gut feeling group - Anova - 2x2 (Coherence x Specificity)

		df	MSE	F	η_p^2	*p*
Specificity	1	115	0.644	1.383	0.012	0.242
Coherence	1	115	0.124	0.176	0.002	0.676
Specificity:Coherence	1	115	0.124	3.002	0.025	0.086

Interaction non-significant

Table 16- Anova Only human face targets in the Sure response group - 2x2 (Coherence x Certainty)

		df	MSE	F	η_p^2	*p*
Specificity	1	114	0.751	1.531	0.013	0.219
Coherence	1	114	0.173	1.735	0.015	0.190
Specificity:Coherence	1	114	0.173	1.279	0.011	0.261

Non-significant

Table 17- Anova Only human face targets in the Coherent stimuli - 2x2 (Certainty x Specificity)

		df	MSE	F	η_p^2	*p*
Specificity	1	229	0.449	0.000	0.000	0.998
Certainty	1	229	0.449	23.545	0.093	0.000
Specificity:Certainty	1	229	0.449	5.329	0.023	0.022

Here we also have the same significant interaction with a similar direction as the one the main text.

Table 18- Anova Only human face targets in the Incoherent stimuli - 2x2 (Certainty x Specificity)

		df	MSE	F	η_p^2	*p*
Specificity	1	229	0.396	0.046	0.000	0.830
Certainty	1	229	0.396	33.729	0.128	0.000
Specificity:Certainty	1	229	0.396	0.554	0.002	0.457

Interaction non-significant

Processing math: 100%
